# The microbiota and metabolome dynamics and their interactions modulate solid-state fermentation process and enhance clean recycling of brewers’ spent grain

**DOI:** 10.3389/fmicb.2024.1438878

**Published:** 2024-09-12

**Authors:** Yueqin Xie, Dongyun Liu, Yang Liu, Jiayong Tang, Hua Zhao, Xiaoling Chen, Gang Tian, Guangmang Liu, Jingyi Cai, Gang Jia

**Affiliations:** Key Laboratory for Animal Disease-Resistance Nutrition of China, Ministry of Education, Institute of Animal Nutrition, Sichuan Agricultural University, Chengdu, Sichuan, China

**Keywords:** Brewer’s spent grain waste, metabolome-microbiome interactions, solidstate fermentation technology, multi-strain inoculation, environmental factor analysis

## Abstract

The massive yield of brewers’ spent grain (BSG) waste inevitably threaten environmental health. Here, solid-state fermentation (SSF) technology featuring multi-strain (MS) inoculation and high-throughput sequencing technology were employed to facilitate the sustainable and clean recycling of BSG waste while revealing the associated underlying microbiological and metabolic mechanisms. MS inoculation displayed a lower pH value (3.91 vs. 4.12) and neutral detergent fiber content (446.24 vs. 476.23 g/kg DM), a higher levels of lactic acid (86.64 vs. 33.07 g/kg DM), acetic acid (6.13 vs. 4.87 g/kg DM), propionic acid (2.78 vs. 2.18 g/kg DM) and crude protein (307.5 vs. 289.15 g/kg DM) than those in the control group. Moreover, MS inoculation inhibited the formation of non-protein-N and ammonia-N, and spoilage microorganism resuscitation, while enhanced substrate preservation. Microbiologically, during the SSF, the group treated with MS inoculation exhibited an increase in the relative abundance of *Leuconostoc* (0.58%∼6.60%), *Weissella* (6.22%∼15.42%), *Enterococcus* (3.15%∼9.08%), *Bacillus* (17.63%∼31.29%), *Lactobacillus* (12.89%∼8.29%), *Pseudoalteromonas* (12.87%∼16.29%), and a decrease in the relative abundance of *Acinetobacter* (0.79%∼0.02%) and *Enterobacteriaceae* (0.78%∼0.24%). Metabolically, starch and sucrose metabolism, arginine and proline metabolism, and phenylalanine metabolism significantly influenced the quality of extruded BSG fermented by MS during SSF. The examination of the correlation between the microbiota, metabolites, and fermentation parameters revealed that complex interactions between microbes and the environment factors impact metabolite production. Collectively, inoculating with MS improved fermentation quality and stability, facilitated the clean recycling of BSG, which is linked to complex interactions among microbes, the environment factors and metabolite production.

## 1 Introduction

Beer is the fifth most consumed beverage worldwide, after tea, coffee, sodium bicarbonate, and milk (Eliopoulos et al., 2022). The annual beer production is increasing continuously, stimulated by economic development and the transition of consumption structure, especially in China. At present, the beer industry has become an important field with the most potential and development prospects in China’s beverage industry, with an annual production of 35.555 million kiloliters in 2022 (Fan et al., 2024). However, a substantial amount of waste is generated during the brewing process, with the primary by-product being brewers’ spent grain (BSG) constituting 80–85% of the total waste produced (Jayant et al., 2018). The annual production of BSG surpasses 30 million tons, with each hectoliter of beer resulting in 15–20 kg of BSG (Radosavljević et al., 2018). It is reported that BSG wastes pose an environmental hazard as they demand around 30–60% of oxygen for complete oxidation (Hang et al., 1975). And BSG waste is rich in essential nutrients, making it an ideal substrate for harmful microorganisms. This can lead to groundwater pollution, greenhouse gas emissions, and pathogen proliferatio (Kavalopoulos et al., 2021). Furthermore, the ultra-high demand for livestock feed causes an inevitable conflict between food production levels for humans and livestock, directly placing food security at risk (Herrero et al., 2015). The adverse impacts stemming from these challenges can stimulate the development of new sustainable livestock feed resources (Newbold et al., 2015). Through the lens of cleaner production, transforming BSG waste into livestock feed has the potential to transition from a linear economy to a circular economy, thereby easing the strain on food supply and environmental conservation (de Mello Santos et al., 2022). This strategy aligns with the European Strategy and Action Plan for the Circular Economy in 2015, highlighting its positive response to current environmental initiatives (Eliopoulos et al., 2022).

However, the key issues for converting BSG waste into livestock feed are its high moisture content of around 70% and the anti-degradation barrier formed by high fiber (cellulose, hemicellulose and lignin), which results in nutrient loss and low nutrient efficiency, respectively (Roth et al., 2019). Our previous study demonstrated that drying BSG to 28% moisture and utilizing extrusion pretreatment can effectively address these challenges by disrupting the cellulosic structure and increasing the accessibility of microorganisms (unpublished). Solid-state fermentation (SSF) has been proposed as a feasible approach to recycle waste into livestock feed by employing solid waste as substrates for cultivating microorganisms to enhance the nutritional quality within the solid wastes (Huang et al., 2023). Microbes and their metabolites significantly influence the quality of the final feed in SSF (Kumar et al., 2021). Studies have highlighted that interactions between microorganisms and metabolites impact ecosystem stability and offer valuable insights for predicting ecological functions (Wang et al., 2022). In addition, studying the relationship between the microbiome, metabolome, and fermentation parameters characterizes the specific microbes and metabolites, helping to understand the potential mechanism (Huang et al., 2023; Xu et al., 2021; Gu et al., 2024). Unfortunately, the microbial and metabolite information related to BSG waste recycling, particularly how composite microbial fermentation impacts the quality of BSG waste feed, remains elusive. Therefore, a reasonable hypothesis suggests that the use of multiple strains (*Bacillus safensis* SCYA3, *Candida tropicalis* SCYA4 and *Bacillus subtilis* SCYA6) can enhance fermentation efficiency, minimize nutrient loss, and decrease pollution production during the SSF of BSG waste. So the effectiveness of multi-strain (MS) on fermentation performance, microbial profiles, and chemical composition were evaluated for feasibility (Yan et al., 2024). 16S rRNA gene sequencing and liquid chromatography–mass spectrometry (LC-MS) were used to investigate the dynamic alterations in the microbiome and metabolome of the extruded BSG inoculated with compound fermentation bacteria. Subsequently, the correlation among environmental factors, microbiome, and metabolome were investigated to unveil the underlying regulatory mechanisms. These findings are instrumental in developing a clean SSF system with enhanced performance, reduced pollution, and for the production of high-quality fermentative feed from BSG waste. Additionally, this research contributes to advancing our understanding of strategies to enhance the quality of fermented extruded BSG waste feed.

## 2 Materials and methods

### 2.1 Fermentative materials preparation

Firstly, BSG (Sichuan Nuke Teide Biotechnology Co., Ltd. Chengdu, China) was pretreated by using DSE32 twin-screw extruder with the extrusion parameters of moisture content 27%, extrusion temperature 106°C and screw speed 16 Hz to obtain extruded brewers’ spent grain (EBSG) (unpublished). Subsequently, EBSG was combined with wheat bran (mass ratio, 9:1) to create a fermentation substrate that would establish a stable fermentation system, as determined through preliminary experiments. Furthermore, the fermentation substrate were randomly divided into 24 individual subsamples, including 4 time durations × 2 treatments × 3 replicates. Two treatment groups were established: one as a control without inoculation (Con), and the other receiving inoculation with a multi-strain (MS) combination comprising *Bacillus safensis* SCYA3 (NCBI accession no. PQ138514), *Candida tropicalis* SCYA 4 (CCTCC NO: M20241049), and *Bacillus subtilis* SCYA6 (NCBI accession no. PQ138515). *Bacillus safensis* SCYA3 and *Bacillus subtilis* SCYA6 were cultured in liquid LB medium at 34°C with agitation at 150 r/min on an orbital shaker and *Candida tropicalis* SCYA 4 was cultured in Yeast extract peptone dextrose medium at 30°C with agitation at 150 r/min on an orbital shaker. After the fermentation substrate was prepared and sterile water was added to achieve a feed to water ratio of 1:1 (w/v), the mixed MS was inoculated into the fermentation substrate at a total inoculation size of 15% (v/w). The fermentation conditions of the inoculation treatment group were as follows: *Bacillus safensis* SCYA3, *Bacillus subtilis* SCYA6 and *Candida tropicalis* SCYA4 were mixed in a volume ratio of 3:2:1, the concentration (MS combination) of 1.0 × 10^7^ CFU/mL, total inoculation size of 15% (v/w), temperature of 34°C, and feed to water ratio of 1:1 (w/v). The fermentation conditions of the Con were the same as those of the treatment group except that the total inoculation size of the MS was replaced by sterile water with the equal volume, and other conditions such as fermentation temperature and feed to water ratio remained consistent. Following treatment, each fermentation bag containing 300 g of subsamples was prepared using polyethylene bags sized at 30 cm × 20 cm, equipped with a one-way breathing valve (only exhaust but not intake). These bags were then stored at ambient temperature (34°C), with samples collected after 1, 3, 5 and 7 days of SSF.

### 2.2 Chemical composition, fermentation parameters and microbial profiles

Samples were ground, passed through a 0.3-mm screen, and analyzed for dry matter (DM), crude protein (CP), neutral detergent fiber (NDF) and acid detergent fiber (ADF) according to standard procedures described by Navarro et al. (2018). And the total nitrogen (TN) content was determined by dividing the CP by 6.25 (AOAC, 1990). Additionally, the water-soluble carbohydrate (WSC) was measured according to the method described by Murphy (Murphy, 1958). The sample (20 g) was blended with 180 mL of deionized water for 60 s using a blender, then filtered through 4 layers of cheesecloth and filter paper. The pH of the filtrate was immediately measured using a glass electrode pH meter. The filtrate was centrifuged at 18,000 × g for 15 min and then the supernatant was collected. Subsequently, the levels of lactic acid (LA), acetic acid (AA) and propionic acid (PA) were determined according to the method described by Li et al. (2023b). 10 g fresh sample were mixed with 90 mL of sterile 0.85% sodium chloride solution and shaken at 150 rpm for 30 min to create a homogenate. Subsequently, lactic acid bacteria (LAB), coliform bacteria, and yeast and yeast were counted according to the method of Li et al. (2023a).

### 2.3 Nitrogen distribution assays

The levels of nonprotein nitrogen (nonprotein-N) were calculated from true protein and crude protein using the method described by Licitra et al. (1996) for the precipitate procedure of true protein. The content of ammonia nitrogen (ammonia-N) was determined according to Broderick and Kang’s method (Broderick and Kang, 1980).

### 2.4 DNA extraction, PCR amplification, sequencing and bioinformatics analysis

Total DNA was extracted from fermentative products using the E.Z.N.A.^®^ soil DNA kit (Omega Bio-tek, Norcross, GA, U.S). The DNA content and quality were assessed. The primer pair 338F (5′-ACTCCTACGGGAGGCAGCAG-3′) and 806R (5′-GGACTACHVGGGTWTCTAAT-3′) were used to amplify the V3-V4 regions of the 16S rRNA gene. The polymerase chain reaction was conducted according to standardized research procedures (Tan et al., 2021). The purified and quantified samples were sequenced for 16S rRNA amplicons using the Illumina NovaSeq PE250 platform (Sanshu biotechnology Co., Ltd, Shanghai, China). The raw Illumina FASTQ files underwent demultiplexing and quality filtering prior to analysis with QIIME (version 1.9.1). Simultaneously, the raw FASTQ files were quality filtered with Trichromatic and then merged using FLASH. Following this, OTUs were clustered at a 97% similarity cutoff utilizing UPARS. The taxonomic classification of each 16S rRNA gene sequence was determined using the RDP Classifier algorithm with a confidence threshold of 70% against the Greengenes 16S rRNA databas. The sequences were submitted to NCBI’s Sequence Read Archive for open access (PRJNA1148331). Diversity values for the samples were assessed using rarefaction analysis with the Chao1 and Shannon indexes. To analyze community dissimilarities in different treatments and fermentation time, we calculated unweighted or weighted UniFrac distances and visualized the results with coordinates obtained from principal coordinates analysis (PCoA). Furthermore, we utilized the Linear Discriminant Analysis Effect Size (LEfSe) method to detect the main genera showing differential abundance. Additionally, we also used the Phylogenetic Investigation of Communities by Reconstruction of Unobserved States (PICRUSt) tool to examine functional differences in bacterial community during EBSG fermentation (Fu et al., 2022).

### 2.5 Non-targeted metabolite analysis

The metabolites were extracted following the method outlined by Xiang et al. (2021). To maintain stability and consistency in instrument analysis for LC-MS, quality control samples were created by pooling 10 μL from each sample. The QC samples were inserted and analyzed in every 10 samples using a UPLC-Orbitrap-MS system (UPLC, Vanquish; MS, HFX). The UPLC analysis conditions are based on earlier methods (Liu et al., 2022). The HRMS data were recorded on a Q Exactive HFX Hybrid Quadrupole Orbitrap mass spectrometer with a heated ESI source, employing the Full-ms-ddMS2 MS acquisition methods. The raw MS data were acquired on the Q-Exactive using Xcalibur 4.1 (Thermo Fisher Scientific) and processed with Progenesis QI (Waters Corporation, Milford, USA). All untargeted metabolomic data used in this publication have been deposited to the EMBL-EBl MetaboLights database with the identifier MTBLS2733. The quantified data analysis using the R package, where they were subjected to multivariate data analysis such as principal component analysis (PCA). The significance of each variable in the classification process was assessed through the calculation of its Variable Importance in Projection (VIP) value. Metabolites were identified through KEGG annotation and enrichment analysis, and the results were then mapped to the KEGG Pathway database.

### 2.6 Statistical analysis

The data for fermentation parameters, nitrogen distribution, chemical composition, and microbial profiles were analyzed using a two-way analysis of variance (ANOVA). Statistical difference between the two treatment groups was determined using Student’s t-tests, and the different fermentation times was analyzed through one-way ANOVA followed by Duncan’s multiple-range test. *P* < 0.05 suggest statistically significant differences. The Pearson correlation among the microbiota, fermentation parameters, and metabolites was examined with SPSS 22.0 software.

## 3 Results and discussion

### 3.1 Fermentation parameters during the solid state fermentation

Table 1 shows that there was an interaction between the application of MS and fermentation time on all fermentation parameters. The MS group had a significant decrease in pH value compared to the Con group from days 3 to 7 (*P* < 0.05). Furthermore, the LA levels in both the Con and MS groups increased throughout the SSF process. However, the LA level in the MS group was significantly higher (*P* < 0.05) than that in the Con group from days 3–7. And the MS group showed a significant increase (*P* < 0.05) in AA level from days 3 to 7 when compared to the Con group. No significant difference in PA level was found between the Con and MS groups (*P* > 0.05), except at 5 and 7 days of fermentation. Moreover, the MS group had significantly higher WSC levels than the Con group from days 3 to 7 (*P* < 0.05), with no significant differences in WSC levels observed between the Con and MS groups on day 1 (*P* > 0.05).

**TABLE 1:** Fermentation parameters of EBSG during the solid state fermentation.

Item ^1^	Days	Treatment ^2^		SEM^c^	*P*-value ^4^		
		Con	MS		A	T	A × T
pH	1	5.14^a^	5.10^a^	0.10	< 0.05	< 0.05	< 0.05
	3	4.78^Ab^	4.39^Bb^				
	5	4.62^Ac^	4.13^Bc^				
	7	4.12^Ad^	3.91^Bd^				
LA (g/kg DM)	1	24.22^d^	24.50^d^	4.11	< 0.05	< 0.05	< 0.05
	3	24.17^Bc^	29.91^Ac^				
	5	27.76^Bb^	36.37^Ab^				
	7	33.07^Ba^	86.64^Aa^				
AA (g/kg DM)	1	3.18^c^	3.40^d^	0.20	< 0.05	< 0.05	< 0.05
	3	3.38^Bc^	4.61^Ac^				
	5	3.97^Bb^	5.27^Ab^				
	7	4.87^Ba^	6.13^Aa^				
PA (g/kg DM)	1	1.12^b^	1.17^b^	0.12	< 0.05	< 0.05	< 0.05
	3	1.39^b^	1.41^b^				
	5	1.95^Ba^	2.26^Aa^				
	7	2.18^Ba^	2.78^Aa^				
WSC (g/kg DM)	1	15.56^a^	16.02^a^	0.56	< 0.05	< 0.05	< 0.05
	3	12.45^Bb^	15.32^Aab^				
	5	10.07^Bc^	13.96^Ab^				
	7	9.00^Bc^	11.33^Ac^				

^1^DM, dry matter; LA, lactic acid; AA, acetic acid; PA, propionic acid; WSC, water-soluble carbohydrates.

^2^Con, control; MS, multi-strain. The data with different capital letters in the same row are significantly different (*P* < 0.05). The data with different lowercase letters in the same column are significantly different (*P* < 0.05).

^3^SEM, standard error of means.

^4^A, the application of MS; T, fermentation time; A × T, the interaction between MS and fermentation time.

### 3.2 Microbial cultivation profiles during the SSF

An interaction between the application of MS and fermentation time was observed in the microbial cultivation profiles displayed in Table 2. The MS group showed significant increases in LAB counts from days 3 to 7 compared to the Con group (*P* < 0.05). Additionally, the MS group showed significant reductions in coliform bacteria counts compared to the Con group during the SSF process (*P* < 0.05). Moreover, MS group possessed significantly higher yeast counts than Con from days 3–7 (*P* < 0.05). No significant differences in yeast counts on day 1 were observed between the Con and MS groups (*P* > 0.05).

**TABLE 2:** Microbial cultivation profiles of EBSG during the solid state fermentation.

Item ^1^	Days	Treatment ^2^		SEM ^3^	*P* value ^4^		
		Con	MS		A	T	A × T
LAB (log_10_ CFU/g FM)	1	5.73^b^	5.81^b^	0.11	< 0.05	< 0.05	< 0.05
	3	5.98^Bab^	6.14^Ab^				
	5	6.12^Ba^	6.94^Aa^				
	7	6.25^Ba^	7.22^Aa^				
Coliform bacteria (log_10_ CFU/g FM)	1	3.73^Aa^	3.03^Ba^	0.12	< 0.05	< 0.05	< 0.05
	3	3.56^Ab^	2.62^Ba^				
	5	3.47^Ac^	2.29^Bab^				
	7	3.37^Ad^	2.13^Bb^				
Yeast (log_10_ CFU/g FM)	1	3.83^c^	3.91^b^	0.16	< 0.05	< 0.05	< 0.05
	3	3.89^Bc^	4.59^Ab^				
	5	3.98^Bb^	5.23^Aab^				
	7	4.08^Ba^	6.10^Aa^				

^1^FM, fresh matter; LAB, lactic acid bacteria; CFU, colony-forming unit.

^2^Con, control; MS, multi-strain. The data with different lowercase letters in the same column are significantly different (*P* < 0.05). The data with different capital letters in the same row are significantly different (*P* < 0.05).

^3^SEM, standard error of means.

^4^A, the application of MS; T, fermentation time; A × T, the interaction between MS and fermentation time.

### 3.3 Nitrogen distribution and chemical composition during the SSF

Table 3 demonstrates that the interactions of applying MS and fermentation time were significant for all parameters except ADF content. A higher (*P* < 0.05) CP level than Con was observed in MS from days 3–7. The contents of non-protein-N and ammonia-N were significantly reduced (*P* < 0.05) by MS compared to Con from days 1–7. Additionally, the NDF content of the MS group was lower than that of Con from days 3–7 (*P* < 0.05), with no significant difference observed between them on day 1.

**TABLE 3:** Nitrogen distribution and chemical composition of EBSG during the solid state fermentation.

Item ^1^	Days	Treatment ^2^		SEM ^3^	*P*-value ^4^		
		Con	MS		A	T	A × T
Crude protein (g/kg DM)	1	280.46^b^	282.91^b^	1.79	< 0.05	< 0.05	< 0.05
	3	280.34^Bb^	290.20^Aab^				
	5	285.78^Bab^	295.96^Aa^				
	7	289.15^Ba^	307.54^Aa^				
Nonprotein-N (g/kg TN)	1	659.83^Aa^	640.59^Ba^	23.76	< 0.05	< 0.05	< 0.05
	3	655.63^Aa^	510.88^Bb^				
	5	650.87^Aab^	408.73^Bc^				
	7	637.01^Ab^	366.25^Bd^				
Ammonia-N (g/kg TN)	1	131.51^Aa^	128.25^Ba^	4.73	< 0.05	< 0.05	< 0.05
	3	129.61^Aa^	101.35^Bb^				
	5	127.29^Aab^	80.63^Bc^				
	7	125.67^Ab^	71.41^Bc^				
NDF (g/kg DM)	1	489.68^a^	489.48 ^a^	2.98	< 0.05	< 0.05	< 0.05
	3	486.49^Aa^	471.93^Bb^				
	5	480.09^Aab^	460.61^Bc^				
	7	476.23^Ab^	446.24^Bd^				
ADF (g/kg DM)	1	163.17	162.92	0.38	0.15	0.14	0.77
	3	162.70	162.26				
	5	162.92	160.82				
	7	161.40	159.79				

^1^DM, dry matter; TN, total nitrogen; Nonprotein-N, nonprotein nitrogen; Ammonia-N, ammonia nitrogen; NDF, neutral detergent fiber; ADF, acid detergent fiber.

^2^Con, control; MS, multi-strain. The data with different lowercase letters in the same column are significantly different (*P* < 0.05). The data with different capital letters in the same row are significantly different (*P* < 0.05).

^3^SEM, standard error of means.

^4^A, the application of MS; T, fermentation time; A × T, the interaction between MS and fermentation time.

### 3.4 Microbial community composition during the SSF

**FIGURE 1:**
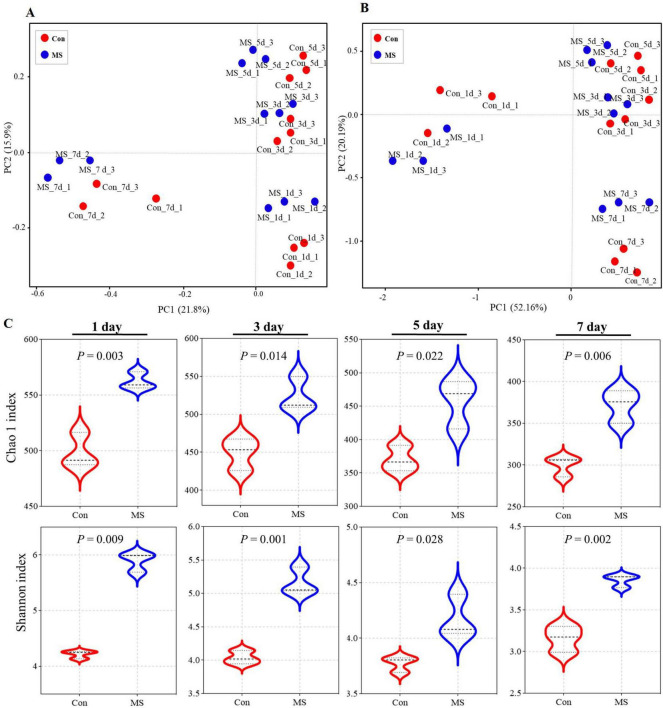
**(A)** The community dissimilarities in different treatments and fermentation time, calculated by unweighted UniFrac distances, with coordinates calculated by principal coordinates analysis (PCoA). **(B)** The community dissimilarities in different treatments and fermentation time, calculated by weighted UniFrac distances, with coordinates calculated by principal coordinates analysis (PCoA). **(C)** The variations of community alpha-diversities (Chao1 richness and Shannon index). Con, control; MS, multi-strain.

The Illumina MiSeq sequencing analysis revealed variations in microbial composition and temporal dynamics during SSF between the Con group and the MS group, highlighting spatiotemporal changes in microbial fermentation. The principal coordinates analysis (PCoA) was used with unweighted and weighted UniFrac distances to determine the factors that shape the differences between EBSG microbiomes (β diversity). The results showed a significant succession of bacterial species over time during fermentation (Figures 1A, B). Three distinct clusters were identified in EBSG fermentation after 1, 3–5, and 7 days. Particularly, the microbial diversity of EBSG fermented for 7 days was distinctly separated from that of the other durations. MS group significantly increased the Shannon index and Chao1 index during fermentation from days 3–7 when compared to the control group in the alpha diversity analysis (*P* < 0.05). However, the bacterial α diversity of both Con and MS groups decreased with the extension of fermentation time (Figure 1C).

**FIGURE 2:**
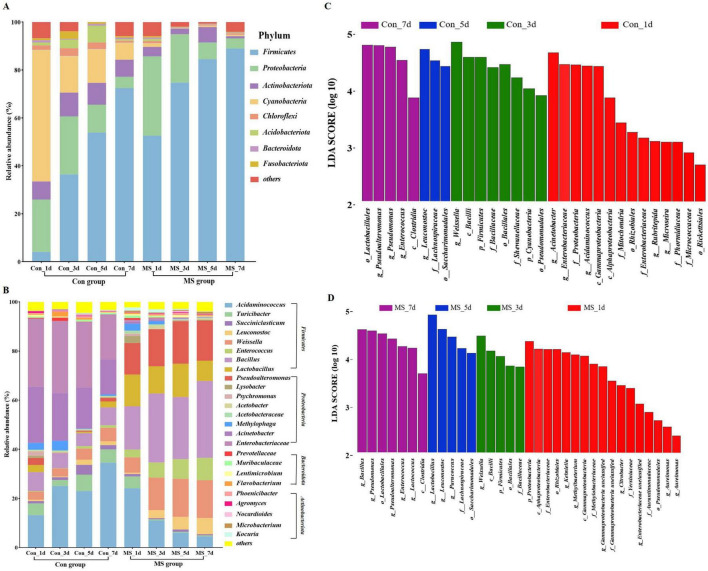
**(A)** Relative abundance of bacteria at the genus level. **(B)** Relative abundance of bacteria at the top 25 species level. **(C)** Linear discriminant analysis effect sizes (LEfSe) analysis of bacterial communities at different fermentation time points in Con group. **(D)** Linear discriminant analysis effect sizes (LEfSe) analysis of bacterial communities at different fermentation time points in MS group. Con, control; MS, multi-strain.

Solid state fermentation is a dynamic process. Conducting microbiological analysis at multiple time points offers a more accurate depiction of microbial dynamics compared to analyzing at a single time point. The microbiota composition dynamics are exhibited in Figures 2A, B. Overall, over eight bacterial phyla were present in each sample (Figure 2A). In the Con group, *Cyanobacteria* and *Proteobacteria* accounted for approximately 76.67% of the sequences at fermentation times of 1 days. As fermentation advanced, *Firmicutes* increasingly became the dominant members, while the relative abundance of *Cyanobacteria* and *Proteobacteria* gradually decreased. While in the MS group, *Firmicutes* increasingly became the dominant members, while the relative abundance of *Proteobacteria* gradually decreased. Continuous variations were detected in the microbial community (genus level) throughout the 4 time points (Figure 2B). *Leuconostoc* (0.58%∼6.60%), *Weissella* (6.22%∼15.42%), *Enterococcus* (3.15%∼9.08%), Bacillus (17.63%∼31.29%), *Lactobacillus* (12.89%∼8.29%), *Pseudoalteromonas* (12.87%∼16.29%) were the dominant bacteria in MS group. The Con group exhibited an ecological imbalance with the dominant bacteria being *Acinetobacter*, *Enterobacteriaceae* and *Acidaminococcus*. While *Acinetobacter* and *Enterobacteriaceae* are types of spoilage bacteria. Interestingly, the abundance of these pathogens was very low in the MS group. These results demonstrated that MS application effectively inhibits the growth of harmful microorganisms in fermented products, enhancing their safety. The LEfSe results showed significant differences in taxonomy between the two groups at different fermentation time points (Figures 2C, D). On day 1, many harmful bacteria, including *Acinetobacter*, *Enterobacteriaceae*, *Proteobacteria*, and *Gammaproteobacteria*, were found in abundance in the Con group.

**FIGURE 3:**
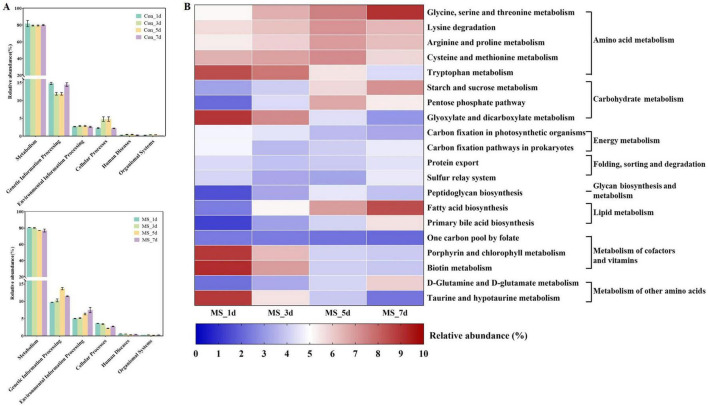
**(A)** Dynamics of bacterial functional profiles during SSF processes analyzed by PICRUSt in level 1 metabolic pathways; **(B)** Dynamics of bacterial functional profiles during SSF processes analyzed by PICRUSt in level 3 KEGG ortholog functional predictions of the relative abundances of the top 20 metabolic functions. Con, control; MS, multi-strain.

The functional changes of bacterial communities in Con and MS groups during different fermentation time shown in Figure 3A were obtained based on the KEGG pathway database. At four time points across 6 metabolic functions, most predicted protein sequences in Con group and MS group ranged from 80.29% to 0.34% and 78.75% to 0.29%, respectively, which represented different pathways. Interestingly, Carbohydrate and amino acid metabolism comprised over 20% of the enriched pathways during SSF, exhibiting significant changes across various time points in the Con and MS groups (Supplementary Figure 1A). It can be revealed that the metabolic interaction between bacteria and compounds, so we speculate that the change in substrate composition during fermentation alters the change of metabolic function of bacterial community. We observed some efficiency differences during SSF at microbial gene function level 3 (Figure 3B and Supplementary Figure 1B), particularly in the MS group (Figure 3B). The majority of genes related to amino acid metabolism (glycine, serine and threonine metabolism, lysine degradation, arginine and proline metabolism, cysteine and methionine metabolism), carbohydrate metabolism (starch and sucrose metabolism) and lipid metabolism (fatty acid biosynthesis) in the MS group showed increasing upregulation during SSF (*P* < 0.05). While the abundances of glycan biosynthesis and metabolism, as well as the metabolism of cofactors and vitamins, showed a decrease.

### 3.5 Metabolomic profiles during the SSF

**FIGURE 4:**
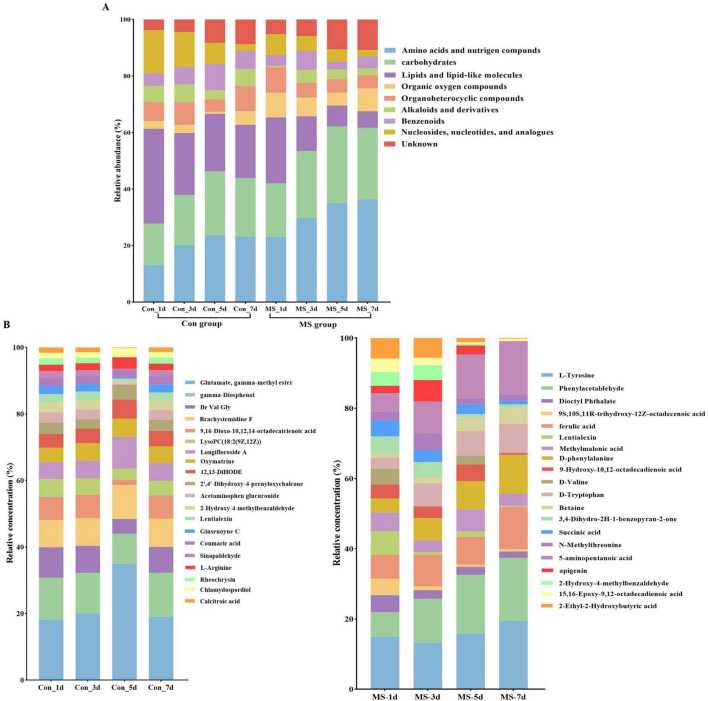
**(A)** Relative compositions of the main metabolites for different fermentation time. **(B)** Top 20 metabolites at different fermentation times. Con, control; MS, multi-strain.

**FIGURE 5:**
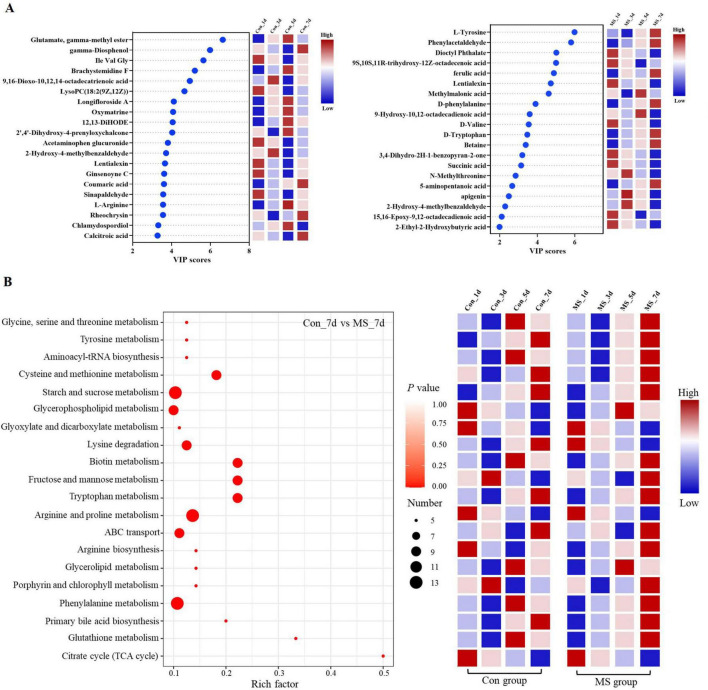
**(A)** A scatter plot of the top 20 distinct metabolites was identified by applying variable importance projection (VIP). **(B)** Enrichment analysis of pathways. Number (dot), number of metabolites annotated to pathways. Con, control; MS, multi-strain.

To assess the metabolome changes in fermented EBSG, we used the untargeted metabolomic approach. A total of 1,517 metabolites were detected across all samples. Unsupervised PCA can reflect variation within and between groups, showing distribution tendency and possible discrete points. PCA with a total cumulative variance of 82.70% comprised of PCA-1 (52.8%) and PCA-2 (29.9%) showed differences in metabolites formation among samples (Supplementary Figure 2). Metabolome analysis indicated that amino acids and nitrogen compounds, lipids and lipid-like molecules, carbohydrates, and nucleosides, nucleotides, and analogues were predominant in Con and MS groups during SSF. Among them, the abundance of carbohydrates, and amino acids, nutrigen compounds during SSF constantly upregulated from days 1 to 5. Contrastingly, the levels of nucleosides, nucleotides, analogs, lipids, and lipid-like molecules constantly declined from days 1 to 7. The MS group showed a higher relative abundance of amino acids and carbohydrates than the Con group at all four time points (Figure 4A). We further analyzed the next level of the top 20 metabolites to identify specific changes in related metabolite (Figure 4B). Figure 5A displayed the VIP values for the top 20 metabolites, indicating their unique metabolic characteristics at various time points following fermentation. Glutamate, gamma-methyl ester, gamma-Diosphenol, Ile-Val-Gly, and Brachystemidine F were the dominant substances in the Con group. While the relative concentration of L-Tyrosine, phenylacetaldehyde, ferulic acid, D-phenylalanine, D-Tryptophan and 5-aminopentanoic acid persistently increased in MS group during the whole fermentation stage.

The pathways were annotated and enrichment analysis was carried out based on the results of differential metabolites. The first 20 metabolic pathways were displayed in Figure 5B. In the MS group, the metabolic pathways most affected during the fermentation process were arginine and proline metabolism, starch and sucrose metabolism, and phenylalanine metabolism when compared to the Con group. On day 1 of SSF, the enrichment of arginine and proline metabolism, and citrate cycle (TCA cycle) decreased throughout the fermentation process. On day 3, the Con group showed significant upregulation of fructose and mannose metabolism as well as porphyrin and chlorophyll metabolism. On day 5 of SSF, the Con group exhibited a notable increase in 6 different metabolic pathways. On day 7 of SSF, the Con group exhibited a notable increase in 7 different metabolic pathways, such as starch and sucrose metabolism, ABC transport and tryptophan metabolism, among others. Whereas, glycerophospholipid metabolism and glycerolipid metabolism were significantly upregulated in the MS group on day 5 of SSF. 16 different metabolic pathways, such as starch and sucrose metabolism, glycine, serine and threonine metabolism, phenylalanine metabolism, tyrosine metabolism, lysine degradation, and tryptophan metabolism etc., were markedly increased in the MS group on day 7 of SSF. The results showed that amino acid metabolism was robust and the quantity of amino acids rose during SSF in the MS group.

### 3.6 The correlations among microbiota, fermentation parameters, and metabolites during the SSF

**FIGURE 6:**
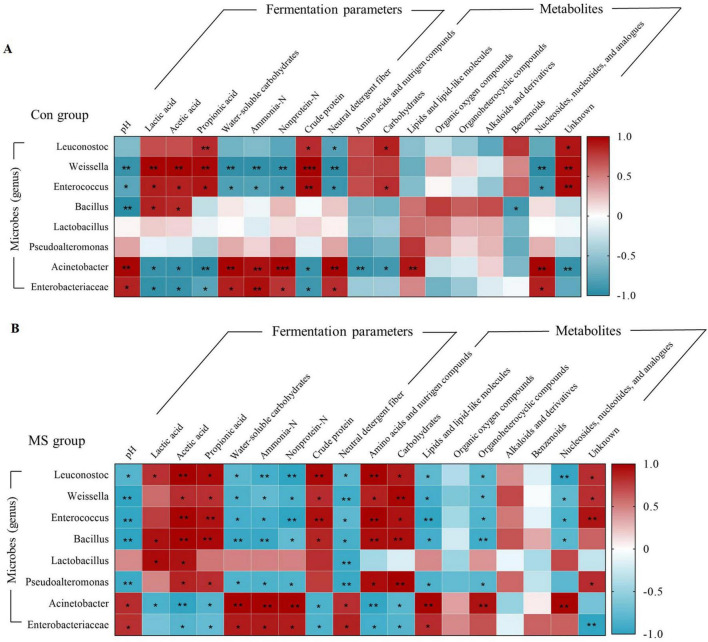
Relationships among the microbiota, metabolites and fermentation quality. **(A)** Relationships among the microbiota, metabolites, and fermentation parameters in Con group during SSF. **(B)** Relationships among the microbiota, metabolites, and fermentation parameters in MS group during SSF. **0.001 < *P* < 0.01, *0.01 < *P* < 0.05, respectively. Con, control; MS, multi-strain.

Correlation analyses were conducted to further investigate the relationship between the changes in microbes (genus level), fermentation parameters, and metabolites (Figure 6A). In the Con group, the relative abundance of *Weissella* and *Enterococcus* showed positive correlations (*P* < 0.05) with LA, AA, PA and CP, and negative correlations (*P* < 0.05) with pH, WSC, ammonia-N, nonprotein-N, NDF and nucleosides, nucleotides, and analogues. While the abundance of *Acinetobacter* and *Enterobacteriaceae* were negatively (*P* < 0.05) related to LA, AA, PA and CP, and positively (*P* < 0.05) correlated with pH, WSC, ammonia-N, nonprotein-N, NDF and nucleosides, nucleotides, and analogues. In the MS group, *Leuconostoc*, *Weissella*, *Enterococcus*, and *Bacillus* showed a significant positive relationship (*P* < 0.05) with AA, PA, CP, amino acids, nutrigen compounds, and carbohydrates. Conversely, they exhibited a negative correlation (*P* < 0.05) with pH, WSC, ammonia-N, nonprotein-N, NDF, lipids, lipid-like molecules, organoheterocyclic compounds, and nucleosides, nucleotides, and analogues. These microbial groups contributed to enhancing the fermentation quality of the substrates (Figure 6B). Furthermore, *Lactobacillus* exhibited a significant positive correlation (*P* < 0.05) with LA and AA, and a significant negative correlation (*P* < 0.05) with NDF. *Pseudoalteromonas* was positively (*P* < 0.05) related to AA, PA, amino acids and nutrigen compunds and carbohydrates, and negatively (*P* < 0.05) related to pH, WSC, ammonia-N, nonprotein-N, NDF, organoheterocyclic compounds, and lipids and lipid-like molecules.

### 3.7 Integrated microbiomic and metabolomic changes in functional pathways during SSF

**FIGURE 7:**
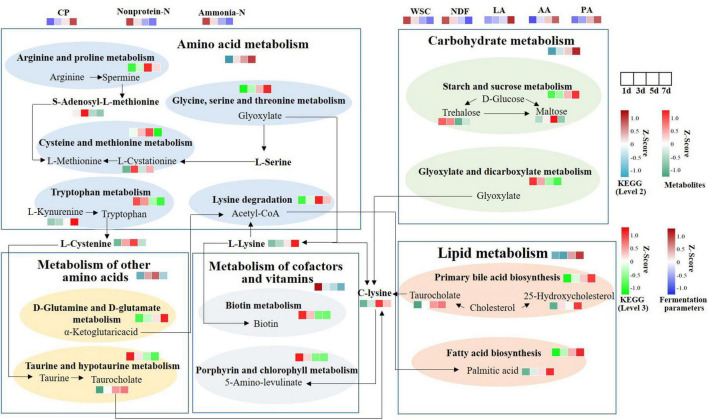
Functional pathway changes of integrated microbiome and metabolomics. KEGG level 2 was selected based on significantly different metabolic data. Using 16S data to predict the abundance of KEGG levels 2 and 3. CP, crude protein; Non-protein-N, non-protein nitrogen; Ammonia-N, ammonia nitrogen; WSC, water-soluble carbohydrates; NDF, neutral detergent fiber; LA, lactic acid; AA, acetic acid; PA, propionic acid.

To further explain the differences of these metabolites resulting from MS inoculation and explore the metabolic mechanism, we integrated microbiomic and metabolomic pathways by combining 16S data and metabolic data (Figure 7). We identified 5 microbial metabolic pathways at level 2 of the KEGG database, which include amino acid metabolism, carbohydrate metabolism, metabolism of other amino acids, metabolism of cofactors and vitamins, and lipid metabolism. Amino acid, carbohydrate, and lipid metabolism increased consistently from days 1 to 7, whereas cofactors and vitamins metabolism decreased. Metabolism of other amino acids were enriched at 5 day. At level 3, glycine, serine and threonine metabolism, D-glutamine and D-glutamate metabolism, primary bile acid biosynthesis, starch and sucrose metabolism, and fatty acid biosynthesis showed a continuous increase in expression. While porphyrin and chlorophyll metabolism, tryptophan metabolism, biotin metabolism, and glyoxylate and dicarboxylate metabolism showed a continuous decrease in expression. Arginine and proline metabolism, cysteine and methionine metabolism, and lysine degradation were enriched at 5 day. C-lysine was the product of amino acid, other amino acids, lipid, and carbohydrate metabolism, and then it could be metabolized by Porphyrin and chlorophyll metabolism to produce 5- amino-levulinic acid. L-cysteine and taurocholate levels were upregulated. And α-ketoglutaric acid was produced from D-Glutamine and D-glutamate metabolism. It can then form Acetyl-CoA through TCA cycle, which was involved in fatty acid biosynthesis.

## 4 Discussion

In the process of SSF, the ideal pH value of fermented feed is 3.8–4.2, which is crucial for inhibiting the metabolism of most undesired microbes and ensuring excellent fermentation quality (Ren et al., 2020). Furthermore, LA with a lower dissociation constant causes a quicker decline in pH value compared to other organic acids (Li et al., 2023b). In this study, the MS group exhibited higher LA concentration and lower pH value, likely attributed to the synergistic antimicrobial effects of metabolites produced by MS inoculation, which led to the suppression of undesirable microbes. Additionally, AA and PA play crucial roles as microbial metabolites in anaerobic fermentation (Li et al., 2023a). MS group showed a significant increase in AA and PA levels, which helped to reduce the pH value of fermentation substrate, thus improving fermentation stability and preventing feed deterioration. Additionally, we also observed a decrease in WSC in both Con and MS groups, indicating that microbial fermentation needs to consume a certain amount of soluble carbohydrates during fermentation, but the level of WSC content in the MS group remained higher than that in the Con group, indicating that MS inoculation enhanced the preservation of fermentation substrates. Collectively, the inoculation of MS boosted fermentative efficiency and improved substrate preservation. The culturable microbial community plays a vital role in evaluating the fermentation efficiency of a fermentation system. Among them, LAB is prominent in the microbiome, indicating successful anaerobic fermentation (Li et al., 2022). Hereon, the MS group displayed high levels of lactic acid bacteria (LAB), suggesting LAB dominance. Coliform bacteria such as *Pseudomonas*, *Enterobacteriaceae*, and *Aeromonas* are consistently associated with energy and dry matter losses (He et al., 2020). The coliform bacteria reduction in the MS group may be attributed to higher levels of short-chain fatty acids or antimicrobial metabolites produced by MS inoculants (Ham et al., 2003). Yeast can thrive in anaerobic conditions and remains unaffected within the pH range of 2–8, leading to increased CP production (Du et al., 2021). However, in this study, MS group showed a high yeast count, which may be due to the change of interaction within the microbial group, which built beneficial conditions for yeast (Li et al., 2022). Hence, the inoculation of MS is beneficial for creating an environment that inhibits the growth of harmful microorganisms.

Assessing fermentative product quality requires careful consideration of nitrogen distribution. Elevated nonprotein-N levels diminish nitrogen availability, resulting in increased nitrogen emissions and environmental worries (Li et al., 2022). The low levels of nonprotein-N in MS can be attributed to the degradation of nonprotein-N-containing substances by microorganisms into ammonia through enzyme activities. Subsequently, the ammonia is further assimilated into amino acids catalyzed by enzyme activities, and these and amino acids are finally metabolized to produce bacterial proteins (He et al., 2019). The increase in CP level provides an explanation for this result. Ammonia-N is a more precise parameter compared to nonprotein-N for reflecting the deamination of amino acids or peptides. Furthermore, ammonia emissions, closely connected to ammonia nitrogen, can potentially transport over long distances in the atmosphere, posing a significant threat to animal and human health, as well as the health of natural ecosystems (Li et al., 2022). As such, the mitigation of ammonia-N levels is a significant national and global concern. Findings from this study show that using MS application can reduce ammonia-N formation. A plausible reason may be that the reduction of undesirable microbes caused by MS treatment is beneficial to reduce the content of ammonia nitrogen. Collectively, MS application can reduce the pollution generation and promote the production of bacterial protein during SSF. Moreover, the inclusion of MS inoculants led to a reduction in NDF levels with no effect on ADF levels. This indicates that the inoculant treatments raised organic acid levels through the degradation of hemicellulose and cellulose (Li et al., 2023b). This results may be attributed to the (hemi)cellulase-producing characteristics of *Bacillus safensis* SCYA3 and *Bacillus subtilis* SCYA6.

Microbial diversity is indicated by the beta and alpha diversity analysis, which represent the richness and uniformity of sample microbiomes (Shang et al., 2022). In this study, the bacterial α diversity of both Con and MS groups decreased with the extension of fermentation time, which may be due to the interaction between metabolic environment and microorganisms, leading to the inhibition of some microbiota in late fermentation (Wang et al., 2023). Additionally, solid state fermentation is a dynamic process. On day 1, many harmful bacteria, including *Acinetobacter*, *Enterobacteriaceae*, *Proteobacteria*, and *Gammaproteobacteria*, were found in abundance in the Con group. The most possible explanation lies in the high pH value in Con group during fermentation (Liao et al., 2023). While *Bacillus*, *Pseudomonas*, *Enterococcus*, *Lactococcus*, *Leuconostoc* and *Weissella* were enriched in MS group. de Gannes et al. (2013) reported that *Bacillus* genus members efficiently degraded protein and cellulose in SSF because of their robust hydrolytic capacity. *Enterococcus* belongs to a mesophilic bacterium that produces LA (Yuan et al., 2018). *Pseudomonas* have been confirmed for their potential role as biocontrol agents to enhance fermentation quality in SSF (Ventorino et al., 2016). *Weissella*, *Leuconostoc*, and *Lactococcus* can produce beneficial metabolites like bacteriocins and exopolysaccharides, as well as enzymes like amylases, proteases, esterases, and glucosidases to break down macromolecules (Zhao et al., 2020). Furthermore, these bacteria play a crucial role in providing flavor precursors for alcohols and esters. Yang et al. (2021) emphasized that the absence of these precursors could lead to a lack of flavor. These dominant genera are a specialized community with characteristic including large-molecule catabolism traits and their ability to produce LA, which is achieved by inoculating with MS. The bacterial structure evolved to show that MS enhanced certain functional bacteria that could establish a symbiotic relationship with the MS. Further, analyzing KEGG gene functions at levels 1 to 3 shows a gradual increase in genes related to carbohydrate and amino acid metabolism during the progression of SSF. Metabolizing cellulose and hemicellulose generates compounds that enhance bacterial growth (Toledo et al., 2017). And amino acids can provide energy and carbon sources for bacteria (López-González et al., 2015). Our findings showed that the degradation of carbohydrates and proteins leads to higher levels of saccharides and amino acids, which are used by the microbial community in fermented EBSG.

The fluctuating metabolite levels during fermentation process are a direct result of microorganisms either producing them or transforming nutrients present in the raw materials used in fermentation. Consequently, the dynamic changes of microorganisms in the fermentation process directly impact the dynamic changes of metabolites. In our study, the MS group showed a higher relative abundance of amino acids and carbohydrates than the Con group at all four time points. The analysis of the top 20 metabolites showed that MS group increased the relative concentration of L-Tyrosine, phenylacetaldehyde, ferulic acid, D-phenylalanine, D-Tryptophan and 5-aminopentanoic acid. Proteins’ nutritional value relies heavily on their amino acid composition, particularly the essential amino acids in animal diets. According to Wang et al. (2022), fermentation has been shown to increase amino acid levels, such as lysine, phenylalanine, isoleucine, and valine. Additionally, phenylamine metabolism is directly linked to the production of flavor compounds during fermentation. Phenylalanine is converted to cinnamic acid by the phenylalanine ammonia lyase, which is further metabolized into various aromatic organic acids by cinnamate 4-hydroxylase. 5-aminopentanoic acid, a biomarker, contributes to the protective and therapeutic effects against hyperlipidemia in animal. The increase in 5-aminopentanoic acid levels indicates that fermentation enhances the potential benefits of the substrates (Zeng et al., 2020). Ferulic acid exhibits prebiotic effects by enhancing the host’s antioxidant capacity and modulating inflammatory responses (Zhang et al., 2022). These results indicate that inoculation with MS improved the production of key flavor compounds and beneficial substances in EBSG fermentation. Metabolites are the final products of an intricate biochemical reaction network that is controlled by different biological processes, such as mRNA, enzymes, genes, and other metabolites. Metabolic pathways, comprising complex metabolic reactions and their regulation, are crucial for understanding metabolic activities and dynamics (Xu et al., 2020). The metabolic pathways analysis showed that amino acid metabolism was robust and the quantity of amino acids rose during SSF in the MS group, including glycine, serine and threonine metabolism, phenylalanine metabolism, tyrosine metabolism, lysine degradation, and tryptophan metabolism. The threonine, phenylalanine, and lysine are essential amino acids, which animals cannot synthesize (Dong et al., 2022). The upregulation of metabolism and the downregulation in degradation of these essential amino acids in fermented EBSG suggest its enhanced nutritional value.

Complex interactions between microbes and the environment factors impact metabolite production. Correlation analyses showed that *Leuconostoc*, *Weissella*, *Enterococcus*, and *Bacillus* showed a significant positive relationship with AA, PA, CP, amino acids, nutrigen compounds, and carbohydrates, while exhibited a negative correlation with pH, WSC, ammonia-N, nonprotein-N, NDF, lipids, lipid-like molecules, organoheterocyclic compounds, and nucleosides, nucleotides, and analogues. It can be seen that *Leuconostoc*, *Weissella*, *Enterococcus* and *Bacillus* were the most dominant genus in the SSF process of MS group. Studies show that *Bacillus* is the main fermentation bacteria, which can promote the natural fermentation of foods rich in protein, produce flavor compounds, and decompose complex food compounds into smaller components during fermentation (Wang et al., 2022). Furthermore, many enzymes secreted by Bacillus can promote the degradation of complex carbohydrates and proteolysis, which also explains the positive correlation between *Bacillus* bacteria and carbohydrates, amino acids, and nitrogen compounds during SSF. *Enterococcus*, *Weissella* and *Leuconostoc* are lactate-producing bacteria that improve taste and flavor by breaking down proteins, fats, and carbohydrates, and by producing aromatic compounds (Yang et al., 2021). The relationships of microbiota, fermentation parameters and metabolites further confirmed the addition of MS promoted the degradation of protein and carbohydrates by *Leuconostoc*, *Weissella*, *Enterococcus* and *Bacillus*, and finally improved the fermentation quality. Finally, based on the results of metabolic pathway integration, we speculate that it may be due to the proliferation of dominant bacteria such as *Leuconostoc*, *Weissella*, *Enterococcus* and *Bacillus* after inoculation with MS, which directly or indirectly participate in amino acid metabolism, carbohydrate metabolism, metabolism of other amino acids, and lipid metabolism, leading to increased metabolic capacity. Consequently, the dominant metabolic function during varying fermentation times after inoculation with MS was further elucidated. The consistency of fermentation parameters and metabolites confirms the metabolic differences resulting from distinct microbial compositions in SSF. However, the detailed metabolic mechanism of the identified metabolites in this study is still not fully unclear, indicating the complexity of the metabolic process of fermented EBSG. In order to address this knowledge gap, future research efforts should prioritize the investigation of secondary metabolites and work towards establishing a standardized metabolic spectrum for fermentation studies.

## 5 Conclusion

Herein, this study systematically studied the dynamic changes of fermentation characteristics, microbial community and metabolites of EBSG during SSF, and revealed that MS inoculation was beneficial in establishing a clean SSF system that is highly efficient, of high quality, and low in pollution. This helps in producing high-quality fermented feed from BSG waste, resulting in low pH and ammonia nitrogen levels, as well as high WSC content. Microbiologically, MS inoculation enhances the competitiveness of *Leuconostoc*, *Weissella*, *Enterococcus* and *Bacillus* in microbial community and promotes metabolic ability. Metabonomics, starch and sucrose metabolism, arginine and proline metabolism, and phenylalanine metabolism are important metabolic pathways that influence the quality of EBSG fermentation by MS. Moreover, studying the correlation among microbial community, metabolites and environmental factors provides valuable clues for constructing an efficient, high-quality and low-pollution fermentation system. The basic fermentation mechanism of obtaining high-quality fermented feed based on BSG waste was guided by exploring the comprehensive changes of microbiology and metabonomics of functional pathways in SSF. Collectively, MS is suitable for the SSF system utilizing EBSG waste, leading to a clean, efficient, high-quality, and environmentally friendly SSF system.

## Data Availability

The original contributions presented in the study are publicly available. This data can be found at the NCBI with accession number: PRJNA1148331.
