# Supplementary material for: The microbiota and metabolome dynamics and their interactions modulate solid-state fermentation process and enhance clean recycling of brewers’ spent grain
Source: Front Microbiol. 2024 Sep 12;15:1438878. doi: 10.3389/fmicb.2024.1438878 (PMC11425715; doi:10.3389/fmicb.2024.1438878)
Supplement: Supplementary file 1 [file Data_Sheet_1.docx]

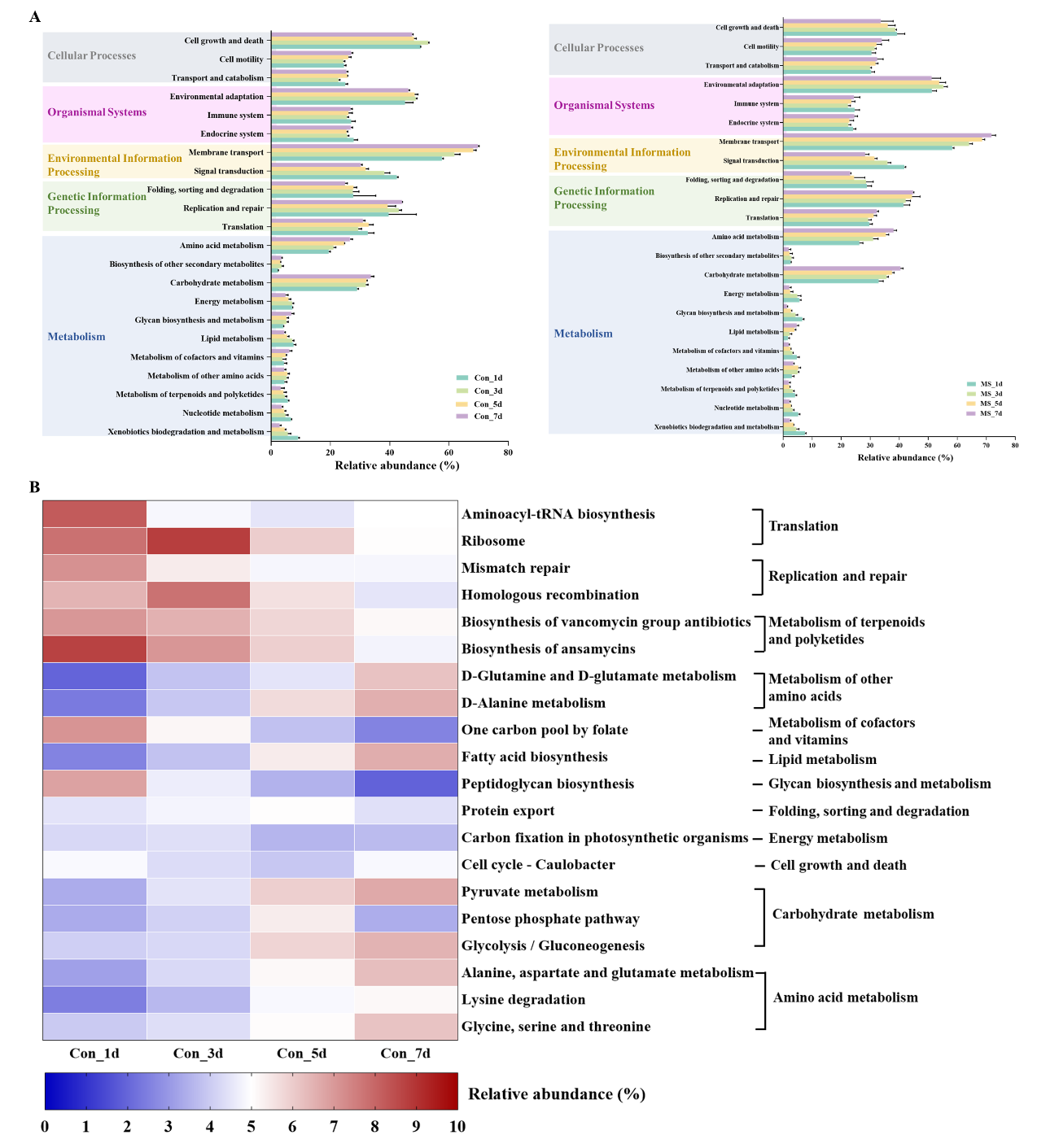


**FIGURE S1.** Dynamics of bacterial functional profiles during SSF processes analyzed by PICRUSt. (A)Level 2 KEGG ortholog functional predictions. (B)Dynamics of bacterial functional profiles during SSF processes analyzed by PICRUSt in level 3 KEGG ortholog functional predictions of the relative abundances of the top 20 metabolic functions. Con, control; MS, multi-strain.


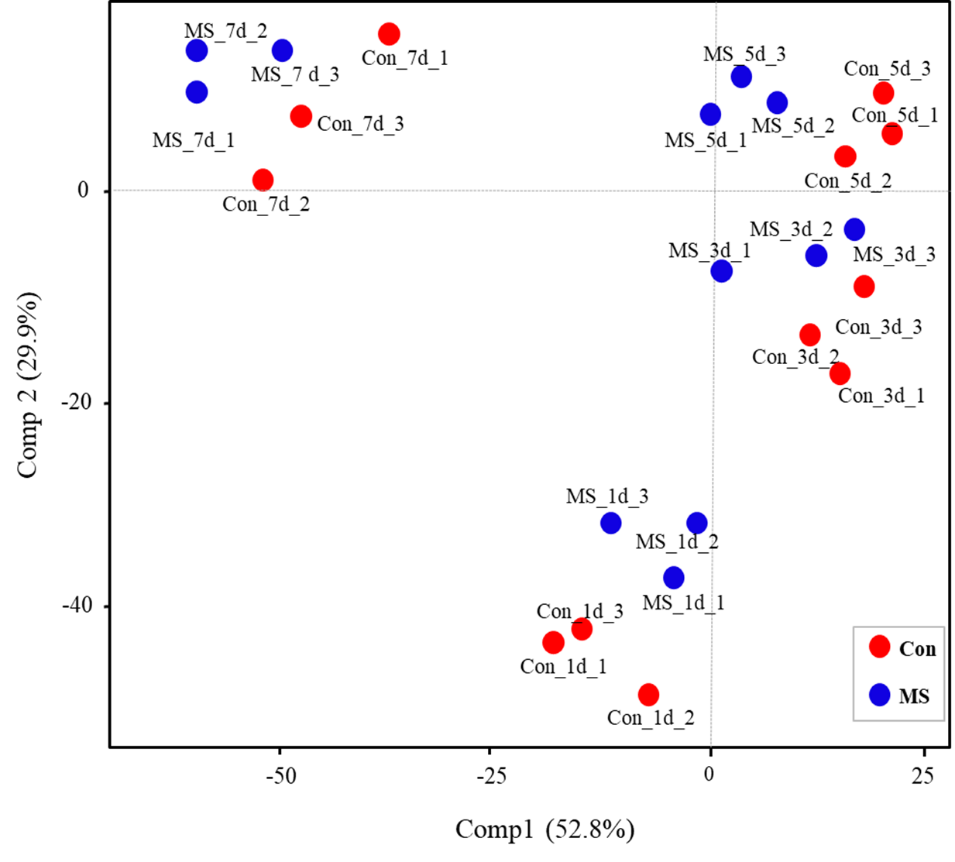


**FIGURE S2.** Principal component analysis (PCA) plot of compounds in fermented EBSG. Con, control; MS, multi-strain.
